# Eustachian Tube Dysfunction in Hearing Loss: Mechanistic Pathways to Targeted Interventions

**DOI:** 10.3390/biomedicines13112686

**Published:** 2025-10-31

**Authors:** Hee-Young Kim

**Affiliations:** 1 Department of Professional, Corporate, and Continuing Education, Harvard Medical School, Boston, MA 02115, USA; hee-young.kim-2022@ppcr.org; Tel.: +82-2-855-7541; 2 Center for Executive and Continuing Professional Education, Harvard T.H. Chan School of Public Health, Boston, MA 02115, USA; 3 Kim Ear, Nose and Throat Clinic, Seoul 08753, Republic of Korea

**Keywords:** Eustachian tube dysfunction, hearing loss, conductive hearing loss, sensorineural hearing loss, mixed hearing loss, otitis media, cholesteatoma, middle ear pressure, Eustachian tube catheterization, artificial intelligence

## Abstract

Hearing loss (HL) affects more than 1.5 billion people worldwide and remains a leading cause of disability across the lifespan. While genetic predispositions, otitis media (OM), and cholesteatoma are well-recognized contributors, Eustachian tube dysfunction (ETD) is an underappreciated but pivotal determinant of auditory morbidity. By impairing middle ear pressure (MEP) regulation, ETD drives conductive hearing loss (CHL) through stiffness and mass-loading effects, contributes to sensorineural hearing loss (SNHL) via altered window mechanics and vascular stress, and produces mixed hearing loss (MHL) when these pathways converge. A characteristic clinical trajectory emerges in which conductive deficits often resolve quickly with restored ventilation, whereas sensorineural impairment requires prolonged, physiology-restoring intervention, resulting in transient or persistent MHL. This review integrates mechanistic insights with clinical manifestations, diagnostic approaches, and therapeutic options. Diagnostic frameworks that combine patient-reported outcomes with objective biomarkers such as wideband absorbance, tympanometry, and advanced imaging enable reproducible identification of ETD-related morbidity. Conventional treatments, including tympanostomy tubes and balloon dilation, offer short-term benefit but rarely normalize tubal physiology. In contrast, Eustachian tube catheterization (ETC) has emerged as a promising, mechanism-based intervention capable of reestablishing dynamic tubal opening and MEP regulation. Looking forward, integration of physiology-based frameworks with personalized diagnostics and advanced tools such as artificial intelligence (AI) may help prevent progression from reversible conductive deficits to irreversible SNHL or MHL.

## 1. Introduction

Globally, more than 1.5 billion people experience some decline in their hearing capacity during their life course, of whom at least 430 million will require care [1]. Among children and adolescents, the burden is substantial, with 134 million affected globally [2]. Recent analyses confirm that otitis media (OM) remains a leading contributor, responsible for long-term auditory morbidity in both pediatric and adult populations [3,4,5].

Traditionally, HL has been divided into conductive, sensorineural, and mixed categories, a framework codified in classic audiological classification systems [6] and reinforced in modern otologic texts that elaborate the structural and functional role of the Eustachian tube (ET) [7]. While conductive forms have often been considered transient and reversible, emerging evidence suggests that Eustachian tube dysfunction (ETD) is a crucial upstream factor contributing not only to conductive loss but also to sensorineural (SNHL) and mixed hearing loss (MHL) [8,9,10].

Historical accounts also reveal that this broader clinical spectrum was appreciated long before modern mechanistic studies. In the mid-19th century, ET obstruction was described as reducing the perception of low-pitched environmental sounds—such as carriage or street noise—anticipating what are now recognized as stiffness and mass effects [11]. These early insights captured both the muffling of lower-frequency resonance and the blunting of clarity for higher-pitched sounds, foreshadowing the dual concepts of stiffness-related and mass-related transmission loss. Later, systematic studies confirmed this frequency-selective pattern, showing that middle-ear effusion imposes inertial loading that preferentially attenuates high-frequency conduction [12]. Building on this trajectory, the clinical model was expanded to include vestibular disturbances, linking middle ear pressure (MEP) imbalance to both auditory and balance functions [13]. Several decades later, “vertigo due to obstruction of the Eustachian tubes” was explicitly characterized as a distinctive clinical entity, underscoring abnormal MEP as a shared substrate for both HL and vestibular morbidity [14]. These early insights foreshadow contemporary perspectives that recognize ETD as a driver of lifelong auditory morbidity.

Mechanistic studies highlight four principal pathways through which ETD impacts auditory outcomes. Negative MEP stiffens the tympanic membrane (TM) and ossicular chain, attenuating low-frequency transmission [15]. Effusion imposes inertial loading on the system, selectively dampening high-frequency conduction [16]. These effects have been validated in vivo through wideband reflectance measurements, which demonstrate frequency-specific absorbance changes under varying MEP [17]. In parallel, experimental studies have shown that pressure differentials across the oval and round windows distort cochlear fluid dynamics, predisposing to vertigo and transient auditory changes [18,19].

Recent technological advances have expanded diagnostic capabilities. Wideband acoustic immittance [20,21], tubomanometry [22], and imaging-based finite-element modeling [23,24] have refined the understanding of ET mechanics. In parallel, artificial intelligence (AI) has emerged as a transformative tool: deep learning enables accurate classification of tympanograms [25], pure-tone audiograms (PTA) [26,27], and otoscopic images of OM [28]. These digital innovations provide unprecedented opportunities to integrate mechanical, physiological, and clinical perspectives on ETD.

This review follows a narrative synthesis approach aimed at integrating historical, mechanistic, and clinical perspectives on ETD and hearing loss. Literature verification was primarily performed via PubMed and Google Scholar.

Additional methodological insights were introduced during the author’s participation in the *Principles and Practice of Clinical Research (PPCR)* program at Harvard T.H. Chan School of Public Health, including discussions on the interpretation of uncontrolled clinical studies.

In parallel, historical perspectives—such as Mudry’s analyses on the evolution of otology—were incorporated to contextualize modern mechanistic understanding.

Furthermore, several references were derived from materials the author had collected and reviewed over years of clinical practice, reflecting questions that arose during patient care and real-world observations. These materials were obtained not only from academic databases but also through professional networks, social-media discussions focused on otolaryngology, and specialty ENT magazines, which often share evolving insights from clinical experience.

This flexible, evidence-informed strategy aligns with the principles of narrative review methodology, allowing integration of historical context, barophysiological mechanisms, and contemporary diagnostic and therapeutic perspectives.

So, the aims of this review are threefold:

(i) to synthesize mechanistic evidence linking ETD to conductive, sensorineural, and mixed hearing loss.
(ii) to evaluate diagnostic and therapeutic strategies in light of these mechanistic pathways, including advances in wideband acoustic immittance, tubomanometry, finite-element modeling, and AI.
(iii) to situate Eustachian tube catheterization (ETC) as the only currently available physiology-restoring, mechanism-based therapy that simultaneously serves diagnostic and therapeutic purposes, thereby embedding it within the broader framework of precision and preventive otolaryngology.

In doing so, this review consolidates over a century of historical insight with contemporary mechanistic, diagnostic, and therapeutic evidence. By reframing ETD as a definitive and modifiable determinant of lifelong auditory morbidity, it highlights the necessity of shifting from symptomatic management to physiology-restoring interventions. This perspective underscores the urgency of embedding ETC and related mechanistic strategies within the emerging paradigm of precision and preventive otolaryngology, ensuring that both historical wisdom and modern innovation converge toward long-term hearing preservation.

## 2. Mechanistic Pathways of ETD Leading to Hearing Loss

### 2.1. Conceptual Framework

ETD does not represent a single disease but a spectrum encompassing structural obstruction, functional failure, barometric dysregulation, and patulous phenotypes. Despite their heterogeneity, all converge on the inability to regulate MEP, which acts as the common pathway imposing pressure differentials across the TM, ossicular chain, cochlear windows, and inner-ear vasculature. These cascades propagate through four mechanistic domains: stiffness effect, impairing low-frequency sound conduction [15]; mass effect, whereby effusion adds inertial loading to the middle-ear system, reducing compliance and selectively dampening high-frequency transmission—a phenomenon observed clinically in otitis media with effusion (OME) [12] and validated experimentally in temporal-bone and wideband reflectance studies [16,20]; window mechanics, altering cochlear fluid dynamics through oval–round window pressure differentials [18,19]; and vascular stress, compromising cochlear microcirculation and strial homeostasis [10,29]. Clinically, these mechanisms manifest not only as conductive loss but also as progressive sensorineural and mixed hearing impairment, often accompanied by vestibular symptoms.

**Figure 1:**
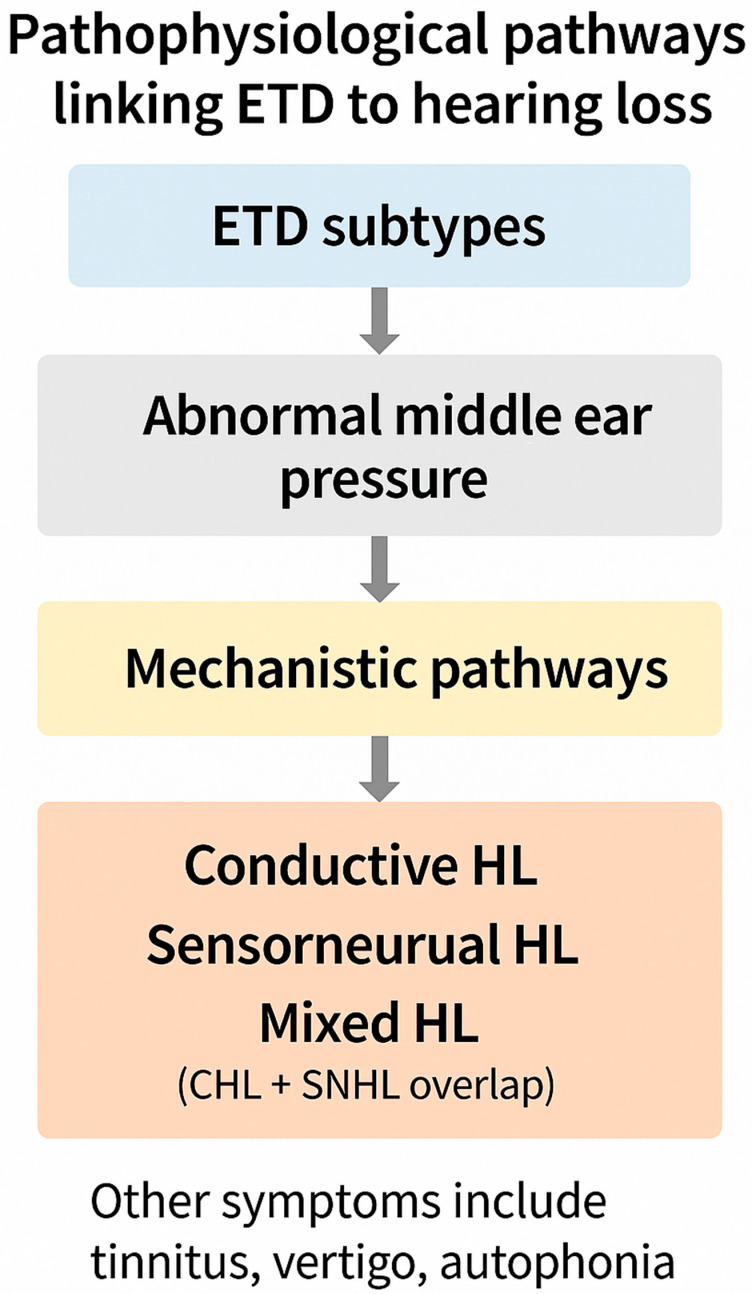
Conceptual framework linking Eustachian tube dysfunction (ETD) to hearing loss.

This integrated framework is schematically illustrated in Figure 1, which depicts how abnormal MEP regulation propagates mechanical and vascular stress across auditory structures. Representative mechanistic studies are summarized in Table 1. Importantly, Table 1 adopts a dual structure, pairing historical “legend” studies that first articulated each mechanistic pathway with contemporary validation studies that experimentally or technologically confirmed those concepts. This contrast underscores not only the deep historical roots of stiffness, mass, window, and vascular mechanisms, but also their modern corroboration through wideband acoustic immittance, finite-element modeling, and other experimental methods. By aligning early conceptual insights with present-day evidence, the table highlights how long-standing clinical observations have matured into validated mechanistic frameworks that continue to shape our understanding of ETD.

Abnormal middle ear pressure, arising from different ETD subtypes, activates four mechanistic pathways: stiffness effect, mass effect, window mechanics, and vascular stress. Together, these mechanisms give rise to conductive hearing loss (CHL), sensorineural hearing loss (SNHL), or mixed hearing loss (MHL, overlapping CHL and SNHL). Additional symptoms frequently include tinnitus, vertigo, and autophonia.

### 2.2. Historical Precursors

The mechanistic framework linking ETD to HL is rooted in observations that long predate experimental validation. Early otologists recognized that disturbances in tubal function could provoke both auditory and vestibular morbidity.

James Yearsley described patients who could not perceive low-pitched environmental sounds such as carriage rumble while still understanding spoken voices, anticipating what is now recognized as the stiffness effect. In the same treatise, he also noted that mucous secretions could accumulate within the tympanum and obstruct the tube, producing a dulling of ordinary sounds—an early anticipation of the mass effect [11].

In 1871, Peter Allen, a British pioneer of otology who was well before his time, mentioned that vertigo due to ETD was relieved by local treatment using an air-douche. Not only that, he also correctly maintained that vertigo is associated with intracochlear pressure (pressure upon the labyrinth fluid) [13].

A major step came when ET obstruction was explicitly reported as a cause of both vertigo and hearing loss, introducing the notion of baro-mechanical ETD [14]. This account underscored that abnormal MEP could act upon the stapes and disturb perilymph dynamics, simultaneously disrupting balance and auditory thresholds, thereby framing ETD as a dual auditory–vestibular disorder.

Attempts at active intervention further highlighted the role of pressure regulation. Politzer’s introduction of “Politzerization” in the mid-nineteenth century aimed to restore ventilation, implicitly recognizing the mass effect of effusion, though later regarded as a crude and sometimes harmful maneuver [35].

By the twentieth century, more objective frameworks emerged. Tympanometric classification established practical correlates for stiffness-related dysfunction in everyday practice [36], while pathological studies revealed degeneration of the stria vascularis and spiral ligament in chronic OM, anticipating the vascular stress pathway and reinforcing that ETD could drive both conductive and sensorineural pathology [10].

Together, these historical milestones demonstrate that the four mechanistic pathways—stiffness, mass, window mechanics, and vascular stress—were anticipated long before contemporary modeling or experimental confirmation.

### 2.3. Conductive Hearing Loss (CHL)

CHL represents the most direct and historically recognized outcome of ETD. The pathophysiology is primarily explained by two complementary mechanisms: stiffness and mass effects.

Stiffness effect. Sustained negative MEP stiffens the TM and ossicular chain, attenuating transmission of low-frequency sounds (<1 kHz). This phenomenon, first described clinically by Yearsley in the 19th century, was experimentally validated in controlled models of acute negative pressure [15]. Wideband acoustic immittance further quantified this effect by showing reduced absorbance in the low-frequency range under negative MEP [17,20]. More recent finite-element modeling extended this understanding by demonstrating how static pressure loading produces progressive microstructural deformation of the middle ear system [31].

Mass effect. In contrast, effusion within the middle ear introduces inertial loading that selectively dampens high-frequency conduction (>2 kHz). Yearsley had already noted that mucous accumulation dulled ordinary sounds, and this insight has been confirmed experimentally: acoustic–mechanical studies demonstrated that fluid reduces high-frequency absorbance in both physical and computational models [12,16]. These findings highlight that ETD’s conductive impact is bidirectional—stiffness impairs low-frequency transfer, while mass effects limit high-frequency transmission.

Clinical vignette. A case of ground-level alternobaric vertigo (GLABV) associated with ETD was reported in 2023. The patient presented with vertigo, recurrent aural fullness, tinnitus, and fluctuating hearing thresholds. Although tympanometry appeared within normal limits, interaural asymmetry of MEP was evident. Following blind transnasal ETC, vertigo resolved alongside tinnitus and ear fullness, and the conductive component of hearing loss rapidly recovered [19].

Synthesis. Collectively, these findings establish that CHL associated with ETD reflects a dual mechanical burden: stiffness from negative MEP and mass from effusion. Both mechanisms can fluctuate dynamically, producing the variable thresholds frequently observed in clinical practice.

### 2.4. Sensorineural Hearing Loss (SNHL)

Although ETD has traditionally been regarded as a purely conductive disorder, substantial evidence indicates that it also contributes to SNHL through abnormal window mechanics and vascular stress.

#### 2.4.1. Abnormal Window Mechanics

Pressure gradients between the oval and round windows can distort perilymph–endolymph dynamics and impair basilar membrane vibration. Experimental studies first established that unequal displacement at the cochlear windows alters inner-ear mechanics [18]. Clinical observations have reinforced this concept: in a case of ground-level alternobaric vertigo (GLABV), interaural MEP asymmetry produced vertigo, tinnitus, and ear fullness, all of which resolved after ETC [19]. More recently, computational modeling further validated that cochlear window distortion represents a robust mechanism of ETD-related inner-ear dysfunction [32].

**Figure 2:**
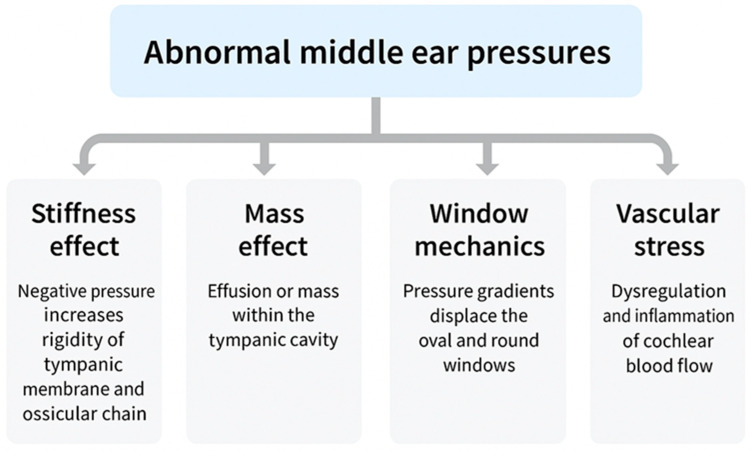
Integrative model of ETD-related hearing loss.

Animal experiments further demonstrated the long-term consequences of sustained pressure dysregulation, showing that surgical obliteration or fistula of the endolymphatic sac in primates produced membranous hydrops [37]. Additional studies confirmed that chronic alterations in perilymph–endolymph balance can expand the endolymphatic space [38,39]. Collectively, these findings suggest that ETD-induced window pressure differentials may not only cause short-term, reversible auditory–vestibular dysfunction but also serve as an upstream trigger for hydrops-like pathology and progressive SNHL. A consolidated overview of these experimental and imaging studies is integrated into the narrative of Section 2.6 and visually summarized in Figure 2.

Clinical vignette 1 [19].

A patient with GLABV presented with vertigo, ear fullness, tinnitus, and fluctuating thresholds. Although tympanometry was within normal limits, interaural asymmetry in MEP suggested a significant pressure imbalance. Following blind transnasal ETC, vertigo resolved alongside auditory improvement. This case illustrates that interaural MEP asymmetry can acutely disturb cochlear fluid dynamics, supporting window mechanics as a reversible pathway in ETD-related SNHL [19].

#### 2.4.2. Vascular Stress

Sustained negative pressure or repeated baro-mechanical stress may compromise cochlear perfusion. Histopathological studies have described ischemic changes in the stria vascularis and degeneration of the spiral ligament in chronic OM [10], and long-term pediatric cohort data further confirm that early middle ear pathology can predispose to progressive SNHL [40]. More recent quantitative work confirmed reduced strial volume and capillary length in human cochleae affected by middle ear disease [41]. These findings suggest that vascular compromise is a critical mediator of ETD-related SNHL.

Clinical vignette 2 (78-year-old patient).

A 78-year-old man, previously prescribed costly hearing aids without tympanometric evaluation, presented with chronic ear fullness and hearing impairment. PTA revealed sensorineural loss without an air–bone gap. Twice-weekly blind ETC was initiated. Within weeks, low-frequency thresholds improved modestly, but high-frequency discrimination remained limited. After three months of continuous treatment, the patient reported perceiving environmental sounds—such as keypad tones and machine beeps—that had previously been inaudible. This delayed yet progressive recovery illustrates how ETC can restore not only conductive function but also cochlear performance through improved window mechanics and vascular stabilization.

Synthesis.

Together, these cases and studies underscore that ETD-related SNHL arises from combined mechanical and vascular stresses at the cochlear level. Importantly, animal models of hydrops demonstrate that chronic pressure dysregulation alone can drive inner-ear pathology, reinforcing the concept that ETD is a potential upstream driver of progressive cochlear dysfunction. Physiology-restoring therapy, such as ETC may therefore achieve both rapid and delayed recovery trajectories by addressing these dual mechanisms.

### 2.5. Mixed Hearing Loss (MHL)

MHL arises when conductive and sensorineural mechanisms overlap, a pattern frequently encountered in chronic or recurrent ETD. As described in Section 2.3 and Section 2.4, stiffness and mass pathways reduce sound conduction, while window mechanics and vascular stress compromise cochlear integrity. In MHL, these processes converge and often potentiate each other: chronic effusion not only imposes inertial loading on the middle ear but may also induce long-term ischemic changes in the stria vascularis and spiral ligament [10,42].

Stiffness effect not only attenuates low-frequency transmission but, when sustained over time, may also provoke irreversible cochlear injury. Pathological and longitudinal studies have demonstrated that persistent negative MEP can contribute to strial atrophy and spiral ligament degeneration, thereby linking stiffness-related ETD to permanent sensorineural decline [10,30,42].

Beyond native auditory decline, ETD-related middle ear pathology may also compromise outcomes in patients receiving cochlear implantation. Case-based and cohort evidence have shown that persistent middle ear disease impairs cochlear implant performance and auditory rehabilitation [43,44,45]. These findings highlight that abnormal middle ear ventilation—even after bypassing the cochlea—remains clinically relevant, underscoring the contribution of ETD to MHL through its impact on both physiologic hearing and implanted devices.

Clinical vignette (illustrative).

A typical case involves a patient with a history of recurrent effusion and multiple tympanostomy tube placements. Audiometry demonstrates both a low-frequency air–bone gap and a high-frequency decline. Following blind transnasal ETC, the conductive component improves rapidly, with closure of the air–bone gap, while the sensorineural component shows only gradual and partial recovery over several months.

This characteristic divergence—rapid reversal of conductive impairment alongside slower, incomplete recovery of sensorineural thresholds—underscores how MHL reflects the cumulative burden of ETD. It also highlights the need for therapies such as ETC that restore physiology and address both middle ear mechanics and cochlear health.

### 2.6. Integrated Mechanistic Perspective

The four mechanistic pathways—stiffness effect, mass effect, window mechanics, and vascular stress—do not act in isolation. Rather, they converge dynamically, producing overlapping clinical phenotypes of conductive (CHL), sensorineural (SNHL), and mixed hearing loss (MHL). Negative MEP may initially stiffen the TM, while effusion imposes inertial loading. At the same time, pressure gradients across the cochlear windows disturb fluid dynamics, and chronic stress compromises strial vascular supply. The clinical outcome depends on which of these processes dominates and how they interact over time.

Importantly, evidence suggests that these pathways may extend beyond transient auditory dysfunction to influence inner-ear pathology. Experimental models have shown that sustained pressure dysregulation can expand the endolymphatic space, producing hydrops-like changes [37,38,39]. Advanced MRI studies have since enabled in vivo visualization of endolymphatic hydrops in patients with Ménière’s disease, linking fluid imbalance to clinical auditory decline [46,47,48]. Although hydrops itself is not the focus of this review, these findings underscore the plausibility that ETD, by chronically disturbing cochlear pressure homeostasis, could act as an upstream driver of inner-ear dysfunction.

This integrative view reframes ETD as more than a transient middle ear disorder. It is instead a lifelong, modifiable determinant of auditory morbidity. By mapping CHL, SNHL, and MHL onto the four mechanistic pathways, clinicians can better predict patterns of recovery and tailor interventions. Representative mechanistic studies are summarized in Table 1, while Supplementary Table S1 consolidates the core experimental reports that directly link ETD to hearing loss. The broader implications of pressure-related hydrops are incorporated into this framework, emphasizing that successful management requires therapies that restore physiology rather than merely bypass dysfunction. The convergence of these pathways is visually illustrated in Figure 2.

Schematic representation of the four mechanistic pathways linking Eustachian tube dysfunction (ETD) to hearing loss. Stiffness effect (negative middle ear pressure) primarily attenuates low-frequency transmission, while mass effect (effusion) dampens high-frequency conduction. Window mechanics disturb cochlear fluid dynamics through oval–round window pressure gradients, and vascular stress leads to ischemic degeneration of the stria vascularis and spiral ligament. In mixed hearing loss (MHL), these processes converge, producing both conductive and sensorineural deficits with divergent recovery trajectories.

## 3. Clinical Manifestations and Subtypes of ETD

### 3.1. Symptom Spectrum

Patients with ETD most commonly present with auditory symptoms reflecting impaired MEP regulation, many of which directly contribute to HL.

- Fluctuating HL—Alternating stiffness and mass effects cause variable thresholds, sometimes shifting from day to day. Negative MEP stiffens the TM and ossicles, impairing low-frequency transmission, whereas effusion introduces inertial loading that selectively dampens high-frequency conduction [15,16,20,31].
- Tinnitus—Frequently reported in ETD and plausibly linked to disturbed window mechanics or vascular compromise. Histopathological studies have shown strial and spiral ligament pathology in chronic middle ear disease [47], while quantitative analyses confirmed vascular compromise in human cochleae [41]. Clinical reports further suggest that tinnitus may improve after physiology-restoring interventions such as ETC, with short-term and long-term recovery patterns [19].
- Auditory–vestibular overlap—Vertigo, disequilibrium, and aural fullness often accompany auditory decline when interaural pressure asymmetry disturbs cochlear fluid mechanics or compromises vascular supply. These manifestations underscore that ETD is not confined to conductive deficits but can also mimic or exacerbate SNHL and MHL patterns [18,19,23,49].
- Autophonia—Characteristic of patulous ETD, this symptom does not itself produce HL but illustrates the broader range of tubal dysfunction [50].

Collectively, these manifestations emphasize that ETD is a multisystem disorder, but its most clinically significant burden lies in fluctuating CHL, SNHL, and MHL. This symptom framework is illustrated in Figure 2.

### 3.2. Structural Obstruction

Structural obstruction arises when hypertrophic adenoids, mucosal edema, or nasopharyngeal masses narrow the ET lumen, preventing its normal opening during swallowing or yawning [7,51]. This impaired ventilation generates sustained negative MEP, which stiffens the TM and ossicular chain, reducing low-frequency transmission efficiency [15,20,31]. At the same time, impaired clearance promotes effusion accumulation, introducing inertial mass loading that selectively dampens high-frequency conduction [12,16]. These stiffness and mass effects frequently coexist, accounting for fluctuating CHL in affected patients.

Beyond conductive pathways, chronic obstruction may also transmit abnormal mechanical stress to cochlear windows and compromise vascular homeostasis, predisposing to progressive auditory decline [10,49]. This reinforces that structural ETD, although originating in the nasopharynx, can extend its pathological influence into both middle-ear and inner-ear compartments.

Clinical vignette. A 45-year-old patient with mucosal edema presented with CHL, tinnitus, and aural fullness. Tympanometry showed a flat type B curve consistent with effusion. Partial improvement occurred with intranasal steroids, but definitive recovery of thresholds was only achieved after ETC. Restoration of tubal aeration alleviated stiffness and mass loading, eliminated tinnitus, and stabilized auditory thresholds.

### 3.3. Functional Obstruction

Functional obstruction occurs when the ET is structurally patent but fails to open effectively during physiologic maneuvers such as swallowing or yawning. Neuromuscular dysfunction of the tensor veli palatini and levator veli palatini muscles, or inhibitory influences from inflammatory conditions such as allergic rhinitis and laryngopharyngeal reflux, are common contributors [9,52,53].

Mechanistically, incomplete or ineffective opening results in intermittent or fluctuating negative MEP. This dynamic stress alters TM compliance and displaces the ossicular chain, producing recurrent stiffness effects at low frequencies. At the same time, pressure gradients across the oval and round windows disturb perilymph–endolymph dynamics, consistent with the window mechanics pathway. Experimental and computational studies have demonstrated that such gradients impair basilar membrane vibration and cochlear homeostasis [19,23,32,51]. Clinical observations further highlight that even in the absence of effusion, functional obstruction can produce tinnitus, fluctuating hearing thresholds, and vertigo [19].

Clinical vignette. A 42-year-old patient reported recurrent fluctuating hearing loss and tinnitus. Tympanometry repeatedly showed type C patterns despite a normal endoscopic appearance of the nasopharyngeal orifice. Blind transnasal ETC directly confirmed impaired opening, and repeated sessions improved both tubal ventilation and long-term symptom stability.

### 3.4. Pressure Dysregulation (Baro-Challenge and GLABV)

Pressure dysregulation represents a distinct mechanistic pathway by which ETD produces both auditory and vestibular morbidity. In normal physiology, balanced middle-ear pressure ensures symmetric transmission across the oval and round windows. When the Eustachian tube fails to equalize barometric changes—whether under rapid ambient pressure shifts (baro-challenge ETD) or during ordinary daily activities—abnormal pressure gradients develop. These gradients disturb cochlear fluid dynamics, displace the oval and round windows, and can precipitate transient sensorineural hearing loss (SNHL), tinnitus, or vertigo [18,23].

A striking clinical manifestation of this mechanism is Ground-Level Alternobaric Vertigo (GLABV), in which impaired tubal opening produces asymmetric window stress even without altitude change. GLABV exemplifies how baro-challenge physiology can extend to everyday settings, resulting in combined vestibular and auditory symptoms. Reports have described this as a “distinct clinical entity,” reflecting historical recognition of Eustachian tube obstruction as a trigger for syndromes uniting hearing loss and vertigo [14]. Recent case-based observations confirm its clinical relevance [19], while experimental and modeling studies provide mechanistic validation [32].

Thus, pressure dysregulation—whether in classical baro-challenge scenarios or in GLABV—serves as a unifying framework linking ETD to inner ear stress. This pathway highlights that ETD is not limited to conductive hearing impairment but can precipitate reversible inner-ear dysfunction through window mechanics and pressure imbalance. As illustrated in Table 1 and supported by the core evidence in Supplementary Table S1, pressure dysregulation provides both a mechanistic and clinical bridge in the spectrum of ETD-induced hearing loss.

### 3.5. Patulous ETD

Patulous ETD occurs when the tube remains abnormally patent at rest, producing autophony, echoing, and barrel resonance. Common causes include weight loss, peritubal fat atrophy, and neuromuscular weakness [7,50]. Unlike obstructive subtypes, patulous ETD does not primarily reflect abnormal middle ear pressures. Instead, the persistently open lumen transmits self-generated sounds such as voice or breathing directly to the middle ear, creating a distinctive auditory distortion.

Clinical studies have reported cases in which patulous ETD developed following significant weight loss, with TM excursions observed to occur synchronously with respiration [54]. These findings underscore the direct anatomical and functional consequences of persistent tubal patency. Differential diagnosis is also essential, since autophony may arise in other disorders such as superior semicircular canal dehiscence, which shares overlapping symptoms but arises from a distinct mechanism [55].

Cross-reference. As illustrated in Figure 2, most ETD subtypes converge on pressure-driven pathways (stiffness, mass, window mechanics, vascular stress). Patulous ETD is unique in that it bypasses these mechanisms, instead producing abnormal sound transmission due to persistent tubal patency. This distinction highlights the heterogeneity of ETD while reinforcing the need for subtype-specific diagnostic and therapeutic approaches.

### 3.6. Integrated Pathophysiological Perspective

Although differing in etiology, all ETD subtypes converge on the same mechanistic pathways—stiffness, mass, window mechanics, and vascular stress. Negative MEP may initially stiffen the TM, while effusion imposes inertial loading. At the same time, pressure gradients across the cochlear windows disturb fluid dynamics, and chronic stress compromises strial vascular supply. The clinical outcome depends on which of these processes dominates and how they interact over time.

Clinical vignette (composite).

A 52-year-old patient reported long-standing aural fullness, intermittent tinnitus, and disequilibrium. Audiometry revealed fluctuating thresholds with both low-frequency conductive and high-frequency sensorineural components. Tympanometry was alternately normal and type C, depending on visit timing. After repeated sessions of Eustachian tube catheterization (ETC), aural fullness and tinnitus resolved, and thresholds stabilized, illustrating how multiple mechanistic pathways—stiffness, mass, window mechanics, and vascular stress—can converge within a single clinical presentation and respond to physiology-restoring therapy.

This integrated perspective emphasizes that ETD is not a transient ventilatory inconvenience but a lifelong, modifiable determinant of auditory and vestibular morbidity. The convergence of overlapping symptom patterns highlights the need for mechanism-based diagnosis and physiology-restoring interventions. Figure 2 illustrates this overlap and links symptom clusters to mechanistic pathways, preparing the ground for the diagnostic framework outlined in Section 4, where diagnostic tools are evaluated in terms of their ability to capture these overlapping processes.

## 4. Diagnostic Pathways in ETD and HL

### 4.1. Transition from Mechanisms to Diagnostics

The mechanistic pathways outlined in Section 2 emphasize that ETD is not a benign ventilatory inconvenience but a driver of progressive auditory and vestibular morbidity. Translating these mechanisms into clinical practice requires accurate diagnosis. Yet, no single gold standard exists. Clinicians must therefore rely on a multimodal strategy that integrates symptom reports, physiologic testing, imaging, and emerging digital modalities [9].

A recent framework distinguishes ETD not only by symptom patterns but also by underlying endotypes—structural, inflammatory, restrictive, or muscular—highlighting the heterogeneity of disease expression [56]. This shift underscores that conventional diagnostic tools, though indispensable, must be interpreted within the broader mechanistic context.

### 4.2. Clinical Symptomatology and Patient-Reported Outcomes (PROMs)

The diagnostic process for ETD begins with recognition of its characteristic symptom constellation. Patients most frequently report aural fullness, fluctuating hearing thresholds, tinnitus, autophonia, vertigo, and disequilibrium, each reflecting abnormal regulation of MEP and its downstream mechanical or vascular effects [7,19]. Although these manifestations are non-specific, they remain indispensable as the initial clinical signal warranting further physiologic testing.

To standardize assessment of symptom burden, several patient-reported outcome measures (PROMs) have been introduced. The Eustachian Tube Dysfunction Questionnaire (ETDQ-7) is the most widely applied instrument, comprising seven Likert-scale items that quantify symptom frequency and severity [57]. Its responsiveness to interventions such as balloon dilation has established clinical utility, yet its validation is largely limited to selected populations, and correlation with objective physiology is modest. Broader generalizability, therefore, remains uncertain.

Complementary PROM tools—including visual analog scales for ear fullness or tinnitus, and disease-specific quality-of-life instruments—offer additional granularity but share the inherent limitation of subjectivity [9]. Recent work emphasizes that PROMs must be interpreted in conjunction with physiologic measures such as tympanometry, wideband acoustic immittance (WAI), or dynamic barometric testing, to avoid misclassification of fluctuating ETD as either normal or irreversible pathology [56,58].

From a clinical perspective, PROMs should thus be viewed not as standalone diagnostics but as first-line screening tools that guide the need for confirmatory physiologic or imaging studies. When aligned with mechanistic pathways—stiffness, mass, window mechanics, and vascular stress—PROM data can enrich diagnostic accuracy by linking subjective experience with objective dysfunction.

### 4.3. Conventional Diagnostic Tools

Traditional assessments remain central to the evaluation of ETD, but each method has important limitations.

Pure-tone audiometry (PTA). PTA is routinely employed to detect CHL, providing quantitative thresholds across frequencies. However, it cannot reliably differentiate whether the loss originates from middle-ear mechanics or inner-ear pathology [15,42].

Impedance testing and tympanometry. Tympanometry remains the cornerstone of clinical ETD diagnosis. Jerger’s classification (Types A, B, and C) provides a simple framework for identifying stiffness-related dysfunction [36]. Type B curves typically reflect effusion or advanced negative pressure, while Type C curves indicate dynamic retraction under subatmospheric MEP. Despite its utility, tympanometry provides only a static “snapshot” of middle-ear status and may miss intermittent or fluctuating dysfunction, particularly in baro-challenge ETD [9].

Eustachian Tube Dysfunction Questionnaire (ETDQ-7). As a PROM, ETDQ-7 complements physiologic testing by quantifying symptom burden. It has been validated in adults undergoing balloon dilation and correlates moderately with tympanometric findings [57]. However, its reliance on subjective recall limits specificity, and generalizability remains constrained outside balloon dilation cohorts [56].

Clinical vignette. A 38-year-old patient presented with recurrent aural fullness and fluctuating tinnitus. Despite classic symptoms, tympanometry consistently demonstrated a normal Type A curve, and pure-tone thresholds were within normal limits. However, during episodes of barometric stress, symptoms worsened markedly. Blind transnasal ETC was performed, resulting in immediate relief of fullness and tinnitus. This case illustrates how conventional tools may fail to capture dynamic dysfunction, and how physiology-restoring intervention can confirm diagnosis in real time.

Taken together, these conventional tools remain indispensable but insufficient. Their sensitivity and specificity vary across subtypes of ETD, and their main limitation is the inability to capture dynamic fluctuations in tubal function, leading to underdiagnosis in patients who present with “normal” tympanograms or borderline audiograms despite persistent symptoms. These limitations highlight the need for physiology-based diagnostic approaches that can directly assess MEP regulation in real time.

### 4.4. Advanced and Emerging Modalities

To address the limitations of conventional tools, advanced diagnostic modalities have been introduced:

Sonotubometry: Introduces a nasopharyngeal sound stimulus and detects transmission during swallowing. Early work demonstrated its utility as an objective measure of tubal opening [59]. Later studies confirmed its ability to detect intra-individual changes in ET ventilatory function and its relevance for patients with a history of middle ear disease [60,61].

Tubomanometry: Applies controlled nasal pressure while recording middle-ear responses [22].

Endoscopy: Enables direct visualization of the ET orifice; newer classifications link orifice morphology to dysfunction [56]

Wideband acoustic immittance (WAI): Provides frequency-resolved absorbance profiles; validated in stiffness and mass effects [20,62].

Imaging (CT, MRI, OCT): Provides structural and sometimes functional information. Histopathological and translational studies demonstrate vascular compromise under chronic MEP stress [47,63]. CT and finite-element modeling further clarify pressure-induced window displacements [31,32]. Emerging imaging tools such as optical coherence tomography (OCT) offer unprecedented resolution for real-time assessment of TM and middle-ear pathology, enabling early detection of reversible ETD-related abnormalities and refining diagnostic precision [64].

AI-enhanced diagnostics: Applies machine learning has been applied to tympanograms, audiograms, and otoscopic images [25,28]. However, without embedding middle-ear physiology, algorithms risk misclassifying reversible ETD-related HL as irreversible SNHL [65]. Emerging imaging tools such as OCT can detect nanometer-scale structural changes of the TM in vivo, offering unprecedented resolution for early diagnosis [66].

Digital biomarkers: Continuous monitoring of middle-ear compliance, baro-sensitivity, and PROM-integrated diaries is under investigation [65].

Together, these modalities mark a transition toward dynamic, multimodal, and physiology-informed diagnostics, highlighting the ongoing shift from static tests toward real-time functional evaluation. Recent systematic reviews have synthesized diagnostic pathways for ETD, emphasizing the heterogeneity of current practice and the relevance of comorbid nasal disease [67].

### 4.5. Vestibular Function Testing in ETD Context

Vestibular function tests (VFTs)—including caloric testing, video head impulse test (vHIT), vestibular-evoked myogenic potentials (VEMPs), and posturography—are frequently performed in patients presenting with vertigo or disequilibrium. However, interpretation must be carefully contextualized. Abnormal or asymmetric middle ear pressure (MEP) can distort VFT outcomes by altering stapes loading, thereby mimicking labyrinthine pathology rather than revealing primary vestibular disease.

Experimental and histopathological evidence support this mechanistic link. Pressure differentials across the oval and round windows have been shown to disturb cochlear fluid dynamics [18,23], while strial and spiral ligament pathology in chronic middle ear disease underscores vascular contributions [41,49]. More recently, finite-element and translational human studies have confirmed that asymmetric pressure loading can directly propagate to auditory–vestibular dysfunction [32].

Clinically, GLABV illustrates this mechanism in a striking way: interaural asymmetry of MEP alone—without altitude change—can provoke disabling vertigo, fluctuating auditory thresholds, and tinnitus. Its recognition as a distinct clinical entity highlights the translational value of window mechanics as a diagnostic model of ETD-related inner ear stress [14,19].

Taken together, these findings underscore that vestibular testing in ETD patients cannot be interpreted in isolation. Instead, results must be integrated with pressure-sensitive measures such as tympanometry, WAI, and endoscopy, to distinguish true vestibular pathology from ETD-induced artifacts. As summarized in Table 1 and Table S1, pressure dysregulation provides both mechanistic and clinical anchors linking ETD to auditory–vestibular morbidity.

### 4.6. Integrated Therapeutic Perspective

No single diagnostic tool suffices for the accurate characterization of ETD. Instead, a multimodal pathway is required—one that combines patient-reported outcomes, physiologic measures, imaging, and emerging digital technologies. Conventional tools such as audiometry and tympanometry establish baseline auditory and middle-ear status, but they provide only a snapshot of function and often miss dynamic or fluctuating disease [9,36]. Patient-reported outcomes, particularly the ETDQ-7, contextualize symptom burden, yet they remain subjective and lack strong correlation with objective physiology [51,57,58].

Advanced methods such as sonotubometry, tubomanometry, and endoscopic evaluation directly assess tubal opening dynamics and orifice morphology, while imaging modalities (CT, MRI, OCT) extend evaluation to structural and vascular correlates of disease. Histopathological and quantitative temporal bone studies confirm that chronic MEP dysregulation can lead to degeneration of the stria vascularis and spiral ligament, reinforcing vascular compromise as a key mechanism [41,42,47,68]. These findings strengthen the vascular stress hypothesis and underscore that ETD can contribute not only to conductive but also to progressive sensorineural hearing loss.

AI now offers the possibility of integrating these multimodal datasets into dynamic, physiology-informed diagnostic frameworks. However, without embedding MEP physiology, AI-driven audiology risks misclassifying reversible ETD-related losses as irreversible cochlear pathology [65].

ETC exemplifies the intersection of diagnosis and therapy. By restoring the tubal opening in real time, ETC functions not only as a treatment but also as a diagnostic probe, directly testing the reversibility of auditory and vestibular deficits. Because ETC relieves this pressure load, it may also act as a protective intervention, potentially preventing progression from reversible conductive patterns to irreversible sensorineural decline [7,19,42,47,65,66,67,68,69,70].

**Figure 3:**
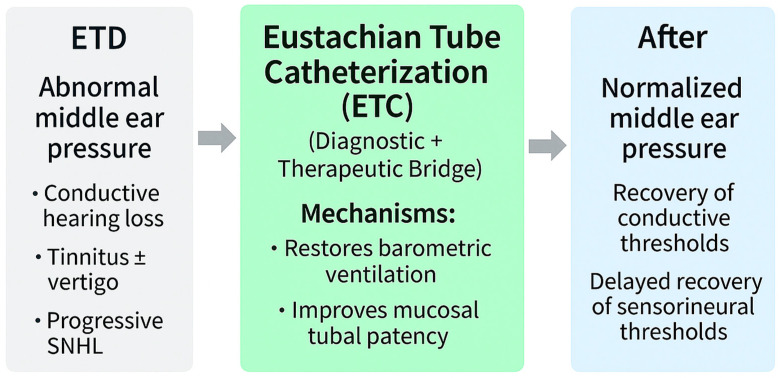
Eustachian tube catheterization (ETC) as a physiology-restoring intervention. By reactivating tubal opening and closure, ETC directly normalizes middle ear pressure, thereby correcting stiffness, mass, and window mechanics pathways. These effects translate into recovery of conductive thresholds. Sustained normalization of pressure may also secondarily mitigate vascular stress, potentially stabilizing progressive sensorineural outcomes. This framework highlights how a single physiology-based therapy spans both immediate and long-term mechanistic domains of ETD-related auditory morbidity.

To ensure cumulative learning and translational precision, diagnostic strategies must be interpreted in the context of mechanistic pathways. Table 1 summarizes representative studies for each pathway. Table S1 consolidates the 13 core mechanistic reports linking ETD to hearing loss. Together with Figure 3, these resources illustrate how conventional tools, PROMs, advanced physiologic modalities, and AI-based monitoring can be integrated into a unified diagnostic strategy that anticipates and guides mechanism-based therapy.

## 5. Therapeutic Landscape of ETD in HL

### 5.1. Transition from Diagnosis to Therapy

The mechanistic pathways outlined earlier—stiffness, mass, window mechanics, and vascular stress—show that ETD is not a benign ventilatory inconvenience but a driver of progressive auditory morbidity. Translating these insights into clinical practice highlights that therapy must be judged not only for symptomatic relief but also for its capacity to target these mechanistic pathways, with the ultimate goal of preserving or restoring hearing across conductive, sensorineural, and mixed domains. Interventions that bypass the tube or palliate symptoms without addressing underlying physiology are limited, whereas therapies that restore tubal barophysiology offer truly targeted potential [9].

Histopathological analyses of human temporal bones have also demonstrated cochlear pathology associated with chronic otitis media and proloned ETD, reinforcing the vascular-stress mechanism underlying progressive auditory decline [71]. Nasopharyngeal and laryngopharyngeal reflux (LPR) exemplify this link between systemic and local pathology. Reciprocal causal relationships between reflux and ETD have been described, with refluxate-induced mucosal inflammation contributing to tubal obstruction [72]. More recently, a prospective cohort highlighted that proton pump inhibitors may alleviate ETD symptoms in LPR-positive patients, reinforcing reflux management as a therapeutic adjunct in ETD care [73]. This paradigm is reflected in the 2019 Clinical Consensus Statement, which formally recognized balloon dilation of the Eustachian tube (BDET) as an accepted intervention for adult obstructive ETD persisting for ≥3 months [74]. The consensus underscored consistent improvements in patient-reported outcomes and tympanometric normalization, while acknowledging limited and inconsistent audiometric gains and insufficient long-term evidence. By contrast, ETC uniquely seeks to restore dynamic tubal physiology rather than mechanically bypassing dysfunction. In doing so, it represents a mechanism-based strategy more directly aligned with hearing preservation across conductive, sensorineural, and mixed domains.

### 5.2. Conventional Medical Therapy

Medical therapy remains the most accessible and widely used first-line approach for ETD. Commonly prescribed agents include intranasal corticosteroids, antihistamines, and proton-pump inhibitors, which aim to reduce mucosal edema and control inflammatory contributors such as allergic rhinitis and laryngopharyngeal reflux [7,9]. These agents can improve nasal and nasopharyngeal conditions, indirectly influencing tubal patency and reducing effusion recurrence.

Reflux-targeted therapy has gained increasing attention. Reciprocal causal relationships between nasopharyngeal/laryngopharyngeal reflux and ETD have been reported, in which refluxate-induced inflammation narrows the tubal lumen [72]. Clinical observations further suggest that proton pump inhibitors may improve ETD symptoms in patients with documented reflux, providing supportive though indirect evidence for reflux control as part of ETD management [73].

Despite these pathophysiologic links, evidence for sustained audiometric improvement remains weak. Randomized controlled trials, particularly in pediatric OME, have shown only modest short-term benefits, with no consistent effect on long-term conductive or sensorineural hearing outcomes [8,75]. Observational and consensus data similarly suggest limited impact on recurrence or progression [52].

Mechanistic alignment: may reduce effusion formation (mass effect) but does not directly address stiffness, window mechanics, or vascular stress.

Targetedness: supportive, non-targeted; provides symptomatic relief but does not modify the pathophysiology of hearing loss.

### 5.3. Tympanostomy Tubes

Tympanostomy tube insertion remains the most frequently performed otologic surgery in children and a cornerstone of management for chronic OME. By bypassing the ET, tubes provide direct middle-ear ventilation, rapidly correcting CHL caused by effusion and restoring thresholds within days [75]. This intervention reduces the developmental risk of language delay and cognitive sequelae in pediatric populations.

Meta-analyses confirmed that tubes significantly improve hearing in the short term, though the effect diminishes after extrusion, and repeated procedures are often required [76]. The Clinical Practice Guidelines by the American Academy of Otolaryngology–Head and Neck Surgery provided standardized recommendations, emphasizing careful selection of candidates, monitoring for complications, and avoiding overuse in children unlikely to benefit [77,78].

Documented risks include tympanosclerosis, persistent perforation, and secondary infections, which increase with repeated insertions. A Cochrane Review further confirmed that while tubes improve short-term hearing outcomes, they do not prevent effusion recurrence or alter long-term language or auditory development, with overall evidence quality rated as moderate to low [79].

Mechanistic alignment: directly bypasses the mass effect of effusion, alleviating conductive impairment.

Targetedness: partially targeted; effective for short-term CHL but not physiology-restoring. Tubes do not influence window mechanics or vascular stress pathways, leaving risks of progressive SNHL or MHL unaddressed.

### 5.4. Balloon Eustachian Tuboplasty (BET)

Over the past decade, BET has been widely promoted as a therapeutic option for ETD. The procedure involves inflating a balloon catheter within the cartilaginous ET to mechanically widen the lumen and facilitate improved ventilation [80,81]. Early uncontrolled studies reported favorable outcomes, including symptom relief and improved patient-reported scores [80,81]. However, uncontrolled or observational studies in other otologic interventions have historically generated overly optimistic impressions of efficacy, as they tend to underreport failures and lack proper comparators [82]. BET appears to follow a similar trajectory.

Subsequent prospective trials suggested improvements in ETDQ-7 scores and middle-ear ventilation parameters [83]. Nevertheless, the overall certainty of evidence remains limited. Several studies have demonstrated inconsistent improvements in tympanometric outcomes and objective physiologic measures [84]. More critically, a recent double-blinded randomized controlled trial with sham intervention found no significant differences between the BET and control groups in either symptom resolution or tympanometric normalization at 12 months [85]. These findings substantially weaken confidence in BET’s long-term efficacy and underscore the need for larger, sham-controlled RCTs with extended follow-up. Contemporary systematic reviews reaffirm that balloon Eustachian tuboplasty is generally safe and yields favorable outcomes in chronic obstructive ETD, though long-term comparative data remain limited [86].

The safety profile of BET is generally favorable, with few reported complications, although isolated cases of barometric or pressure-related adverse events have been documented [87]. Regulatory approval in several regions, including clearance by the U.S. FDA in 2016, has accelerated adoption despite the persisting uncertainty regarding long-term outcomes [88].

Mechanistic alignment: primarily targets structural obstruction, potentially mitigating stiffness-driven CHL.

Targetedness: partially targeted; while effective in relieving obstruction, BET does not address window mechanics or vascular stress, and evidence for cochlear protection remains lacking.

### 5.5. Eustachian Tube Catheterization (ETC)

ETC, though historical in origin, remains a uniquely physiology-restoring intervention. Unlike tympanostomy or balloon Eustachian tuboplasty (BET), which bypasses or mechanically dilates the lumen, ETC directly reactivates the dynamic act of tubal opening and closing, thereby normalizing MEP regulation itself [69].

In my clinical practice, ETC is performed primarily as a blind, air-based procedure under topical anesthesia while the patient remains seated. This approach restores normal tubal physiology by gently insufflating air through the catheter to reopen the lumen and normalize middle-ear pressure without mechanical dilation or mucosal trauma. Although the same catheter can be used to deliver topical agents—such as steroids or decongestants—in selected cases, such applications are relatively uncommon. Air insufflation alone is generally sufficient to restore tubal function and relieve symptoms. Compared with endoscopic techniques, the blind method avoids patient discomfort and potential injury from the simultaneous insertion of both a scope and a catheter. This clarification reflects the author’s clinical experience across thousands of procedures, emphasizing the physiological rather than purely mechanical principle of treatment.

Immediate outcomes include reversal of negative pressure, restoration of aeration, and recovery of low-frequency thresholds attributable to relief of stiffness-related transmission loss [15]. These findings align with wideband acoustic immittance studies showing pressure-dependent absorbance consistent with stiffness mechanics [89].

Beyond conductive restoration, case-level and histopathological evidence suggest gradual recovery of auditory thresholds, tinnitus improvement, and partial reversal of sensorineural hearing loss [34,49]. Clinical observations further demonstrate resolution of baro-related vertigo and fluctuating hearing after ETC, underscoring its dual role as both a diagnostic probe and therapeutic modality [19].

Catheterization techniques were advanced in the early nineteenth century, with Nicolas Deleau (1799–1862) providing the first systematic case reports linking Eustachian tube dysfunction to hearing loss, tinnitus, vertigo, and emesis [90]. Adam Politzer (1835–1920) popularized the procedure through systematic teaching and widespread clinical use [35,91]. James Yearsley (1805–1869) introduced the concept of “stomach deafness” as a reflux-mediated cause of obstruction [11]. Peter Allen (1833–1870) recognized that middle-ear pressure dysregulation could disturb both cochlear and labyrinthine function [13]. Frederick W. Merica (1889–1950) later defined vertigo due to tubal obstruction as a distinct clinical entity, while also emphasizing its concomitant impact on hearing function [14]. Building on this lineage, Hee-Young Kim has reintroduced Eustachian tube catheterization in the modern era as the only physiology-restoring, mechanism-based therapy, reframing it as a cornerstone of precision otolaryngology [19,58].

Mechanistic alignment: uniquely addresses stiffness, mass, window mechanics, and vascular stress.

Targetedness: the only currently available physiology-restoring, mechanism-based therapy.

Clinical implications: candidate for guideline integration, enabling stratification of patients and long-term hearing preservation.

### 5.6. Symptom-Specific Outcomes

The therapeutic response in ETD is best understood when analyzed by individual symptom domains, as each reflects different underlying mechanistic pathways.

Aural fullness and fluctuating CHL: These symptoms are the most responsive across available therapies. Tympanostomy tubes, BET, and ETC consistently provide rapid relief by restoring middle-ear aeration and reducing stiffness or mass effects. Evidence includes multiple RCTs in pediatric populations for tubes [75,79] and prospective trials for BET [83], though durability varies. ETC provides additional long-term stabilization by restoring tubal physiology rather than bypassing it [19,69].

Tinnitus: Improvement has been reported primarily after ETC, likely reflecting normalization of cochlear window mechanics and vascular perfusion [41,49]. Clinical observations support partial reversibility when MEP dysregulation is corrected [19]. By contrast, medical therapy and BET show inconsistent effects on tinnitus.

Vertigo and disequilibrium: These are especially relevant in baro-challenge ETD. Case series describe resolution after ETC when interaural pressure asymmetry is corrected [19]. GLABV, as a sentinel clinical entity, highlights how window mechanics–driven vestibular stress can reverse once physiological ventilation is restored. Robust controlled trials are lacking, but mechanistic plausibility is strong [32].

Autophony: This symptom, typical of patulous ETD, shows little responsiveness to conventional or physiology-restoring interventions. Supportive management—weight stabilization, positional strategies, and occasional surgical interventions—remains the mainstay [54,55]. Importantly, atypical forms of autophony can also arise in obstructive ETD, emphasizing the need for precise diagnostic differentiation.

Sensorineural hearing loss (SNHL): Partial recovery has been observed following ETC, but improvement is typically gradual and incomplete. These outcomes are consistent with the time required for vascular and cochlear window normalization [19,71]. Longitudinal human studies are needed to confirm whether early physiology-restoring interventions prevent progression from reversible to irreversible SNHL.

Summary:

Symptom-specific outcomes reveal a gradient of responsiveness: conductive symptoms (fullness, fluctuating thresholds) respond rapidly, whereas cochlear and vestibular symptoms (tinnitus, vertigo, SNHL) recover more variably or slowly. Autophony remains a separate challenge, underscoring ETD’s heterogeneity. These patterns emphasize the importance of mechanism-based and physiology-restoring therapies, with ETC standing out as the only intervention that directly addresses all four mechanistic pathways.

## 6. Limitations and Future Directions

### 6.1. Evidence Gaps and Methodological Limitations

Although ETD is increasingly recognized as a driver of hearing loss, the evidence base remains fragmented. Most studies are small, observational, and single-center, limiting external validity. Randomized controlled trials (RCTs) of balloon Eustachian tuboplasty (BET) and medical therapy often rely on subjective outcomes such as the ETDQ-7, with few objective physiologic or audiometric endpoints [83,85]. Long-term durability of benefit is uncertain, and reports of ETC, though promising, are largely confined to case series and require replication in multicenter trials [19,69].

### 6.2. Mechanistic Validation

Mechanistic evidence is uneven across pathways. Stiffness and mass effects are well supported by experimental and clinical studies [15,16,20]. In contrast, evidence for window mechanics and vascular stress remains largely indirect, based on histopathology or modeling [32,47,49]. Direct human studies confirming the reversibility of cochlear dysfunction after restoration of MEP are still lacking.

### 6.3. Diagnostic Standardization

Diagnosis is hampered by the absence of a gold standard. Conventional tympanometry and ETDQ-7 provide useful but incomplete information [9,57]. The traditional type A/B/C classification offers only a static snapshot and may miss intermittent or dynamic dysfunction, leading to false negatives in fluctuating patients [92]. Advanced modalities such as sonotubometry, tubomanometry, and wideband acoustic immittance (WAI) offer improved physiologic resolution but remain heterogeneous in protocol and reproducibility [56]. Imaging approaches, including OCT, provide structural–functional correlation and show promise for dynamic phenotyping, though validation is still limited [64].

### 6.4. Integration of Biomarkers and PROMs

Overreliance on PROMs remains a major limitation. While the ETDQ-7 is widely used, correlations with objective physiologic measures are modest, raising concerns about construct validity. PROM-based endpoints can inflate perceived treatment efficacy when subjective improvement is not matched by durable changes in thresholds or physiology. Integrating PROMs with objective biomarkers such as MEP monitoring, WAI, and OCT is essential to enable mechanistically grounded phenotyping [20,58].

### 6.5. Future Research Priorities

Future research should prioritize multicenter RCTs with standardized endpoints spanning PTA, WAI, and validated PROMs. Mechanistic validation of window mechanics and vascular stress requires translational studies using OCT, computational modeling, and quantitative imaging. PROMs must be anchored against biomarkers to ensure diagnostic accuracy, and longitudinal cohorts are needed to capture delayed recovery trajectories. AI should evolve from image-based classification toward physiology-informed prediction, ensuring that reversible ETD-related sensorineural loss is not conflated with irreversible cochlear pathology [25,28,65].

Within this framework, ETC stands uniquely positioned as the only physiology-restoring, mechanism-based therapy. Rigorous evaluation of its capacity to prevent progression from conductive dysfunction to irreversible cochlear injury could establish it as a cornerstone of precision otology.

## 7. Conclusions

ETD should no longer be regarded as a benign or transient condition confined to pediatric OM. Instead, it represents a lifelong determinant of auditory and vestibular health, contributing not only to conductive impairment but also to progressive SNHL and MHL. By synthesizing historical insights, mechanistic pathways, and contemporary diagnostic advances, this review reframes ETD as a central and modifiable risk factor in otology.

Among available interventions, ETC is uniquely distinguished as a physiology-restoring, mechanism-based therapy. Unlike tympanostomy or balloon dilation, ETC directly reestablishes the dynamic regulation of MEP, thereby addressing the upstream defect that links ETD to auditory and vestibular morbidity. While high-quality trials remain limited, converging evidence from clinical observation, experimental modeling, and historical precedent supports its potential role as a targeted intervention.

Looking forward, precision otolaryngology will require integration of patient-reported outcomes with objective biomarkers, validation of mechanistic pathways, and rigorous multicenter trials. Within this evolving framework, ETC emerges not as an obsolete relic but as a candidate cornerstone of precision otolaryngology—capable of preserving hearing, mitigating vestibular symptoms, and redefining the management of ETD across the lifespan. Incorporation of physiology-restoring strategies into future guidelines could enable stratified, mechanism-based care and ensure long-term preservation of auditory function. Ultimately, recognition of ETD as a modifiable determinant of hearing health reframes clinical practice, emphasizing prevention and physiology rather than palliation.

## Figures and Tables

**Table 1:** Core mechanistic pathways linking ETD to auditory and vestibular outcomes. Historical legends paired with modern validations highlight four mechanistic pathways: stiffness effect, mass effect, window mechanics, and vascular stress.

Mechanistic Pathway	Legend (Conceptual Introduction)	Clinical Development	Experimental Validation	Recent Validation
Stiffness Effect	Yearsley, J. [11]. Deafness Practically Illustrated. London: John Churchill.	Lildholdt, T., et al. [30]. Negative middle ear pressure and hearing loss in children. Scand Audiol, 8: 117–120.	Finkelstein, Y., et al. [15]. Acute negative middle-ear pressure and low-frequency loss. Acta Otolaryngol, 112: 88–95.	Muyshondt, P.G.G.; Dirckx, J.J.J. [31]. Finite-element analysis of pressure-induced stiffness effects. Hear Res, 400: 108116.
Mass Effect	Yearsley, J. [11]. Deafness Practically Illustrated. London: John Churchill. (effusion as dampening sound)	Hunter, L.L.; Margolis, R.H.; Rife, J.P. [12]. High-frequency hearing loss in OME children. Ear Hear, 17: 1–11.	Ravicz, M.E.; Rosowski, J.J.; Merchant, S.N. [16]. Middle-ear fluid mass loading attenuates high-frequency transmission. Hear Res, 195: 103–130.	Feeney, M.P.; Keefe, D.H.; Sanford, C.A. [20]. WAI in OME ears validates mass effect patterns. Ear Hear, 38: 605–613.
Window Mechanics	Allen, P. [13]. Lectures on Aural Catarrh. London: John Churchill. (ET obstruction affects cochlear windows)	Merica, F.W. [14]. Vertigo due to obstruction of the Eustachian tubes. JAMA 118: 1282–1284.	Voss, S.E.; Rosowski, J.J.; Merchant, S.N.; Peake, W.T. [18]. Middle-ear pressure and cochlear mechanics. JASA 100(5): 3388–3398.	Zablotni, R., et al. [32]. Sound-induced round window vibration—experiment and numerical simulations. Appl Sci, 15(1): 301.
Vascular Stress	Wittmaack, K. [33]. Über die Pneumatisation des Schläfenbeines. Jena: Fischer. (vascular compromise theory)	Paparella, M.M.; Goycoolea, M.V.; Meyerhoff, W.L. [10]. Inner ear pathology and otitis media. Ann. Otol. Rhinol. Laryngol., 89: 249–253.	Ishiyama, A., et al. [29]. Temporal bone pathology shows vascular compromise in OM. Otol Neurotol, 27: 361–367.	Bovee, C.M., et al. [34]. Endocochlear potential & vascular perfusion in COM (human). JARO.

## Data Availability

No new data were created or analyzed in this study. Data sharing is not applicable to this article.

## Supplementary Materials

The following supporting information can be downloaded at https://www.mdpi.com/article/10.3390/biomedicines13112686/s1. Table S1: Core human studies related to the Eustachian tube and its mechanistic role in hearing loss (ETD–Human Core 13). All references cited in Table S1 are included in the main reference list.

## Abbreviations

The following abbreviations are used in this manuscript:

AI	Artificial Intelligence
BET	Balloon Eustachian Tuboplasty
CHL	Conductive Hearing Loss
DALY	Disability-Adjusted Life Year
ET	Eustachian Tube
ETC	Eustachian Tube Catheterization
ETD	Eustachian Tube Dysfunction
ETDQ-7	Eustachian Tube Dysfunction Questionnaire (7-item)
GLABV	Ground-Level Alternobaric Vertigo
HL	Hearing Loss
MEP	Middle Ear Pressure
MHL	Mixed Hearing Loss
OCT	Optical Coherence Tomography
OM	Otitis Media
OME	Otitis Media with Effusion
PROM	Patient-Reported Outcome Measure
PTA	Pure-Tone Audiometry
QoL	Quality of Life
RCT	Randomized Controlled Trial
SNHL	Sensorineural Hearing Loss
TM	Tympanic Membrane
VFT	Vestibular Function Test
WAI	Wideband Acoustic Immittance
