# Supplementary material for: Eustachian Tube Dysfunction in Hearing Loss: Mechanistic Pathways to Targeted Interventions
Source: Biomedicines. 2025 Oct 31;13(11):2686. doi: 10.3390/biomedicines13112686 (PMC12650131; doi:10.3390/biomedicines13112686)
Supplement: Supplementary file 1 [file biomedicines-13-02686-s001.zip › biomedicines-3931451-supplementary.pdf]

Table S1. Core human studies related to the Eustachian tube and its mechanistic role in hearing loss (ETD–Human Core 13).

Mechanism	Year	First Author	Study	Key Findings
Stiffness effect	1992	Finkelstein	Finkelstein Y, Zohar Y, Talmi YP, Shapira A. Effects of acute negative middle-ear pressure. Acta Otolaryngol. 1992;112: 88–95. [15]	Acute negative pressure stiffens TM and ossicles, attenuating low-frequency conduction.
	2006	Shahnaz	Shahnaz N, Bork K. Wideband Reflectance Norms for Caucasian and Chinese Young Adults. Ear Hear. 2006, 27, 774–788. [89]	WAI quantified stiffness-related low-frequency loss.
	2021	Muyshondt	Muyshondt PGG, Dirckx JJJ. Finite-element modeling of static pressure loading. Hear Res. 2021;400: 108116. [31]	Modeled microstructural deformation with sustained pressure.
Mass effect	1996	Hunter	Hunter LL, Margolis RH, Rife JP, Giebink GS. High-frequency hearing loss in otitis media with effusion. Ear Hear. 1996;17(1): 1–11. [12]	OME adds inertial mass, selectively dampening high-frequency conduction.
	2004	Ravicz	Ravicz ME, Rosowski JJ, Merchant SN. Middle-ear fluid and high-frequency absorbance. Hear Res. 2004;195:103–130. [16]	Effusion decreases high-frequency absorbance in models.
	2017	Feeney	Feeney MP, Keefe DH, Sanford CA. Wideband acoustic immittance under pressure. Ear Hear. 2017;38:605–613. [20]	Validated stiffness and mass effects in vivo.
Window mechanics	1942	Merica	Merica FW. Vertigo due to obstruction of the Eustachian tubes. JAMA. 1942;118:1282–1284. [14]	Linked ET obstruction to vertigo and hearing loss.
	1996	Voss	Voss SE, Rosowski JJ, Merchant SN, Peake WT. Middle-ear pressure and cochlear mechanics. J Acoust Soc Am. 1996; 100(5):3388–3398. [18]	Pressure gradients distort cochlear window mechanics.
	2023	Kim	Kim HY. Ground-level alternobaric vertigo due to ETD. Cureus. 2023;15(3):e36830. [19]	Case report linking ETD with GLABV and reversible auditory–vestibular symptoms.

	2025	Zablotni	Zablotni R, Tudruj G, Latalski M. Sound-induced round window vibration. Appl Sci. 2025;15(1):301. [32]	Computational and experimental validation of window distortion.
Vascular stress	2006	Ishiyama	Ishiyama A, et al. Unbiased Stereological Estimation of the Spiral Ligament and Stria Vascularis Volumes in Aging and Ménière's Disease Using Archival Human Temporal Bones. J. Assoc. Res. Otolaryngol. 2007, 8, 8–17. [29]	Described vascular compromise in stria vascularis with OM.
	2010	Joglekar	Joglekar S, Morita N, Cureoglu S, Schachern PA, Paparella MM, Adams ME. Cochlear pathology in human temporal bones with otitis media. Acta Oto-Laryngol. 2010, 130, 472–476. [71]	Showed ischemic degeneration in cochlea due to OM.
	2019	Pillutla	Pillutla SVP, Kaur C, Roy TS, Jacob TG. Volume and capillary length of stria vascularis in diseased cochleae. J Microsc Ultrastruct. 2019;7(3): 117–123. [41]	Reduced strial volume and capillary length, supporting vascular stress mechanism.
